# Ethical clearance paper as a bottleneck

**DOI:** 10.11604/pamj.2024.47.198.43491

**Published:** 2024-04-18

**Authors:** Subah Abderehim Yesuf

**Affiliations:** 1 Department of Family Medicine, St. Peter's Specialized Hospital, Addis Ababa, Ethiopia

**Keywords:** Ethical clearance, bottleneck, solutions

## Abstract

The significance of the ethical review process in human-based research undertakings cannot be overemphasized as it is necessary to uphold ethical standards and protect participants. However, the review process per se can act as a bottleneck, potentially hindering research progress and leading to academic dishonesty. The present work explores the benefits and challenges of ethical review, emphasizing issues like intellectual theft, forced authorship, and the stifling of independent researchers. Proposed solutions include leveraging previously approved designs, empowering experienced professors for clearance, establishing panels of researchers, creating voluntary ethical approval offices, utilizing private consultancy offices, and establishing a transnational ethical clearance authority. In conclusion, this work stresses the importance of finding mechanisms to streamline the ethical review process while maintaining ethical standards to foster integrity in research and combat academic dishonesty.

## Perspectives

The ethical review process is an essential component of research endeavors, ensuring that all studies are conducted in a manner that respects the rights and welfare of participants. It plays a crucial role in ensuring the integrity and credibility of research following the ethical standards of the Declaration of Helsinki [1-3]. However, the process of securing ethical approval can be marred by several complex challenges. It can even act as a bottleneck, slowing down the progress of research undertakings and potentially hindering scientific advancement [4,5]. Consequently, it can open avenues for academic dishonesty. The commonly encountered issues such as intellectual theft forced authorship queries, and the blunting of independent researchers highlight the need for a reevaluation of existing ethical review mechanisms. In this work, the author will explore the challenges and benefits of the ethical review process and discuss potential solutions to streamline the process without compromising ethical standards.

**Benefits of the ethical review process:** the ethical review process plays a crucial role in ensuring that research is conducted ethically and responsibly. By requiring researchers to consider the potential risks and benefits of their studies, the review process helps to protect participants from harm and promotes the responsible use of research resources. Additionally, the review process can help to identify potential ethical issues that may not have been apparent to the researchers themselves.

**Challenges of the ethical review process:** the ethical review process can be time-consuming and resource-intensive, with researchers often facing lengthy delays in obtaining approval for their studies. This can be particularly problematic in fields where timely research is critical, such as public health or emergency medicine. Additionally, the review process can be subjective, with different reviewers interpreting ethical guidelines differently and potentially leading to inconsistent decisions.

### Bottlenecks in research works

**Intellectual theft:** one of the significant concerns in research ethics is intellectual theft, where ideas, concepts, or findings are misappropriated without proper attribution. Such instance of unethical practice undermines the originality and credibility of research work, leading to unfair advantages for the perpetrator(s).

**Forced authorship queries:** another common issue is forced authorship, where individuals are pressured or coerced into including undeserving authors in their research papers. This practice distorts the credit and recognition that should be rightfully attributed to those who have contributed significantly to the research.

**Blunting of independent researchers:** stringent ethical review processes can sometimes discourage independent researchers from pursuing innovative ideas or unconventional methodologies due to the fear of facing prolonged approval processes or ethical scrutiny. This can stifle creativity, and diversity and hinder the progress of research in various fields. In short, the prevalence of these issues underscores the urgent need to address ethical review as a potential entry point for academic dishonesty. This “corruption of a different color” poses a significant threat to the credibility and trustworthiness of scholarly work.

### Proposed solutions

**Application of previously approved designs:** one solution to address the bottleneck in research works due to ethical review processes is to allow researchers to apply previously ethically approved designs from reputable institutions or studies without the need for additional approval. This approach can streamline the process for well-established methodologies and reduce unnecessary delays.

**Empowering professors for clearance:** another approach is to empower experienced professors or researchers to provide clearance independently for certain types of research projects (mainly, non-interventional studies) that meet predefined ethical criteria. This delegation of authority can expedite the approval process for straightforward studies and alleviate the burden on institutional review boards while ensuring accountability within academic circles.

**Establishing panels of experienced researchers:** one plausible solution is to create panels of seasoned researchers who can evaluate and approve ethical protocols independently enhance efficiency and uphold ethical standards without institutional constraints.

**Voluntary ethical approval offices:** another viable alternative is creating offices where experienced mentors or professors, including professor emeritus, volunteer to oversee and approve ethical papers can offer a reliable and efficient pathway for researchers. This initiative can help uphold ethical standards while supporting researchers in navigating the complexities of ethical review processes.

**Empowering private consultancy offices:** one potential solution for addressing delays and challenges in securing ethical paper clearance is to outsource this task to authorized consultancy offices. By equipping these offices to provide clearance, the process can be streamlined and expedited.

**Transnational ethical clearance authority:** grant providers could collaborate to establish a transnational authority responsible for granting ethical clearance simultaneously, harmonizing standards across borders and streamlining the approval process.

## Conclusion

The ethical review process is a critical component of research, ensuring that studies are conducted in a manner that respects the rights and welfare of participants. While the process can be a bottleneck, researchers and reviewers must work together to find ways to streamline the process without compromising ethical standards. By implementing solutions such as leveraging previously approved designs and granting clearance authority to qualified individuals, stakeholders can mitigate the challenges posed by ethical review bottlenecks, foster a culture of integrity in research practices, and safeguard against academic dishonesty in its various forms.
